# Blood Flow Restriction Using a Pneumatic Tourniquet Is Not Associated With a Cellular Systemic Response

**DOI:** 10.1016/j.asmr.2021.12.018

**Published:** 2022-02-11

**Authors:** Mark C. Callanan, Hillary A. Plummer, T. Meares Green, Tyler Opitz, Thaddeus Broderick, Nicole Rendos, Adam W. Anz

**Affiliations:** a Orthopedic Clinic, Shreveport, Louisiana, U.S.A.; b U.S. Army Aeromedical Research Laboratory, 6901 Farrel Road, Fort Rucker, Alabama, U.S.A.; c Oak Ridge Institute for Science and Education, Oak Ridge, Tennessee, U.S.A.; d UNC Orthopedics at Goldsboro, Goldsboro, North Carolina, U.S.A.; e Andrews Institute for Orthopedics & Sports Medicine, Gulf Breeze, Florida, U.S.A.; f Florida Bone & Joint Specialists, Gulf Breeze, Florida, U.S.A.; g Emory University, Atlanta, Georgia, U.S.A.

## Abstract

**Purpose:**

The purpose of this study was to determine the effects of blood flow restriction (BFR) using a pneumatic tourniquet on CD34^+^ cells, platelets, white blood cells, neutrophils, lymphocytes, lactate, and glucose compared with standard exercise.

**Methods:**

Fifteen healthy volunteers (8 males and 7 females, 28.6 ± 3.6 years old) who were able to perform the exercise sessions on a VersaClimber participated. Participants were randomized to undergo an experimental (EXP) occluded testing session using the pneumatic tourniquets on all 4 extremities and a control (CON) session. The exercise protocol concluded after 9 minutes or when participants reached a rating of perceived exertion of 20. Blood draws were performed before testing and immediately after the exercise session. Blood analysis consisted of complete blood counts as well as flow cytometry to measure peripheral CD34^+^ counts as a marker for hematopoietic progenitor cells (HPCs).

**Results:**

A significant increase from before to after exercise values was observed in both the EXP and CON groups with CD34^+^, WBC counts, platelets, and lymphocytes; however, no differences existed between EXP and CON groups for any variable. CD34^+^ increased in the EXP (3.1 ± 1.6 vs. 4.3 ± 1.8 cells · L^–1^; *P* < .001) and CON (3.3 ± 1.9 vs. 4.4 ± 1.4 cells · L^–1^; *P* < .001) sessions. White blood cells also significantly increased in both the EXP (7.8 ± 1.4 vs. 11.8 ± 2.5 K · L^–1^ K · L^–1^; *P* < .001) and CON (7.5 ± 1.8 vs. 11.3 ± 3.0 K · L^–1^; *P* < .001) sessions. Platelets also increased in both the EXP (258.6 ± 52.5 vs. 309.9 ± 52.7 K · L^–1^; *P* < .001) and CON (263.1 ± 44.7 vs. 316.1 ± 43.9 K · L^–1^; *P* < .001) sessions, and conversely, a significant decrease in the average neutrophil counts in the EXP (mean difference = –13.7%; *P* < .001) and CON (mean difference = –13.2%; *P* < .001) sessions was observed. Lymphocyte counts in the EXP (mean difference = 22.8%; *P* < .001) and CON (mean difference = 19.3%; *P* < .001) sessions increased significantly.

**Conclusions:**

There were no significant differences in systemic cellular responses when undergoing aerobic-based exercise with and without a pneumatic tourniquet system.

**Level of Evidence:**

2, prospective comparative study.

Exercise with blood flow restriction (BFR) is becoming a popular modality of use for both strength and conditioning as well as orthopedic rehabilitation.^1^, ^2^, ^3^, ^4^, ^5^ Compared with traditional strength training paradigms, BFR is advantageous because it allows for the use of submaximal loads to increase muscular size and strength with less stress placed on the joints.^6^ Systemic cellular responses such as increases in CD34^+^ and cellular expression of genes related to muscle upregulation occur during exercise with BFR, which may contribute to increases in muscular size and strength to proximal muscle groups that are not directly occluded.^7^, ^8^, ^9^, ^10^ The same ability for increases in proximal muscle size and strength have not been demonstrated in matched controls undergoing traditional training methods.^6^ The increases in proximal muscle size and strength with the use of BFR is ideal for orthopedic rehabilitation in patient populations who are unable to perform high-intensity exercise and who have failed to improve with traditional therapy.^2^^,^^5^^,^^11^^,^^12^

BFR devices are essentially pneumatic torniquets that work with either set levels of continuously monitored and controlled levels of occlusion or specific calculated manual occlusion pressures based on participant factors. The occlusion of blood flow provided by commercial BFR devices results in hypoxia to the working tissue that likely leads to a cascade of systemic cellular response that contributes to increased muscle size and strength.^13^ Lactate and growth hormone levels have been shown to increase 0 to 40 minutes after BFR,^14^, ^15^, ^16^, ^17^, ^18^ and metabolic overload from the accumulation of hydrogen and lactate may activate interleukin-6, macrophages, and neutrophils.^19^ BFR has also been shown to induce a local angiogenic response through upregulation of vascular endothelial growth factor, another proposed mechanism for the noted efficacy of BFR therapy.^20^

The amount of blood flow occlusion that is induced may vary between BFR devices and potentially limits the amount of systemic cellular responses that occurs with exercise. If the occlusion provided does not create a hypoxic environment in the working tissues, there may be limited efficacy for increasing muscle size and strength. Most of the scientific literature on the cellular responses to BFR has been performed using pneumatic BFR devices that adjust in real time to ensure consistent limb occlusion pressure throughout the full range of motion of an exercise. These types of devices are more cumbersome and restrictive in their use, and can be expensive. Pneumatic devices, however, have been shown to ensure consistent occlusion is provided throughout the exercise.^21^^,^^22^ In the portable tourniquet system, the cuffs are manually inflated before exercise but the pressure is not monitored or adjusted electronically during the exercise. The portability of the tourniquet system used in the current study makes it advantageous to use in clinical settings; however, there is currently a gap in knowledge regarding its efficacy in creating beneficial systemic cellular responses.

Despite the previously studied mechanisms of efficacy for BFR therapy, the degree of mobilization of the cellular components of blood, including hematopoietic progenitor cells (HPCs), to the peripheral circulation after exercise with BFR using the tourniquet system in this study is unclear. This could be another potential mechanism for the efficacy of BFR, as well as something that could be used to manipulate point-of-care products. The purpose of this study was to determine the effects of BFR using a pneumatic tourniquet on CD34^+^ cells, platelets, white blood cells (WBCs), neutrophils, lymphocytes, lactate, and glucose compared with standard exercise. It was hypothesized that exercise with BFR using pneumatic tourniquets would stimulate a systemic cellular response to increase CD34^+^ cells, platelets, WBCs, neutrophils, lymphocytes, lactate, and glucose that would not be observed during regular exercise alone.

## Methods

All procedures were approved by the hospital’s institutional review board. This study was approved by the Baptist Hospital Pensacola Institutional Review Board (approval 1129783). Before data collection, all testing procedures, risks, and benefits of the specific study were explained to each participant, and written informed consent was obtained. Healthy adults aged 20 to 39 years were recruited to participate. Participants were excluded if they had a history of uncontrolled hypertension, diabetes, autoimmune disorders, blood disorders, disorders requiring immunosuppression, cancer, an ongoing infectious disease, or significant cardiovascular, renal, hepatic or pulmonary disease or used steroids. Furthermore, participants were excluded if they had had an orthopedic injury within the past 6 months. Fifteen healthy adults (8 males and 7 females, 28.6 ± 3.6 years old; 172 ± 11 cm height; 74.3 ± 16.1 kg weight) were enrolled in this study (Table 1). One female participant was removed from the data set due to abnormally high (>2 standard deviations from the mean) pre-exercise complete blood count (CBC) and flow cytometry results, leaving 14 participants in the study. Each participant underwent a standard physical exam performed by a nurse or orthopedic sports medicine fellow, including completion of a medical history and assessment of activity level with the Tegner Activity Level Scale.^23^ Once all screening processes were passed, the participants were enrolled for a testing appointment. Participants were asked to refrain from strenuous exercise for 24 hours and from alcohol and caffeine for 12 hours before each testing session.

**Table 1:** Participant Demographics

Characteristic	Value
Sex	8 M, 7 F
Age (years)	28.6 ± 3.8
Height (m)	1.7 ± 0.11
Weight (kg)	74.3 ± 16.1
Tegner Score	5.5 ± 1.0

Data are mean ± standard deviation.

An a priori power analysis (G∗Power 3.1.9.3) revealed a sample size of 10 participants was necessary to detect large effects (200%) using a power of 0.9 and α of 0.05. Sufficient power has been confirmed on previous mobilization studies.^10^ The sample size of this study was increased to 15 to account for potential participant withdrawal.

**Fig 1:**
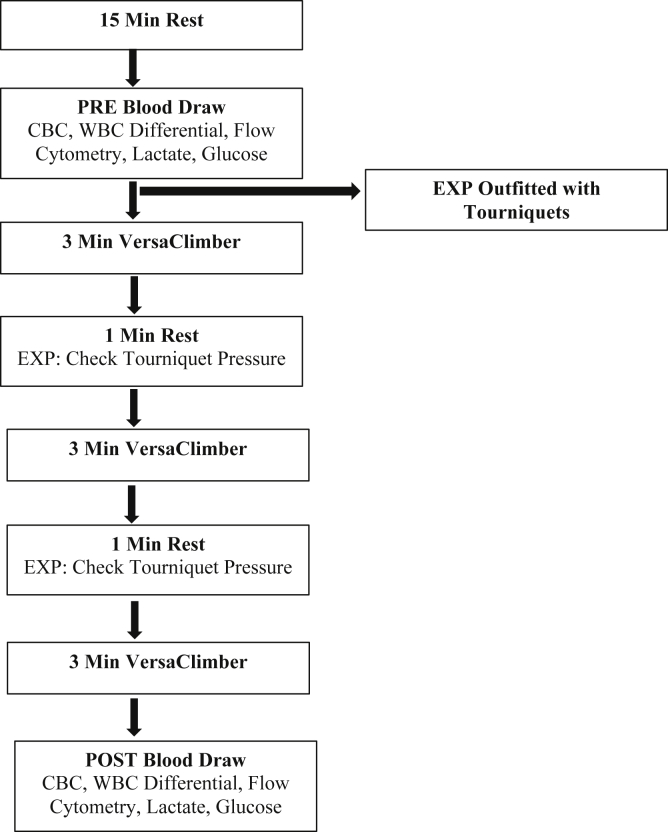
Pneumatic tourniquet exercise session flow chart.

A repeated-measures randomized crossover design was performed with the (B)Strong Training System ((B)Strong, Park City, UT). The exercise protocol is summarized in the Figure 1. Participants rested in the sitting position for 15 minutes before each testing session. A volume of 6 mL of venous blood was drawn from an antecubital vein into two 3-mL blood collection tubes (Vacuette® 454246 Blood Collection Tube, Greiner Bio-One, Monroe, NC) before (PRE) and after (POST) exercise. Three milliliters of whole blood was used to obtain a CBC with a WBC differential using a Sysmex automated hematology analyzer (Sysmex America, Lincolnshire, IL). Flow cytometry (Cytomics FC500 Flow Cytometer, Beckman Coulter Life Sciences, Indianapolis, IN) was used to quantify the amount of CD34^+^ hematopoietic progenitor cells present in the peripheral blood.

Fingerstick capillary samples were used to evaluate blood lactate and glucose levels. A Lactate Plus portable lactate analyzer (Nova Biomedical, Waltham, MA) and Contour® Next blood glucose meter (Ascensia Diabetes Care US, Parsippany, NJ) were used to measure blood lactate and blood glucose, respectively. Fingers were cleaned with an alcohol swab, and then a single-use lancet was used to puncture the finger for blood testing. Both sides of the puncture site were pressed gently as needed to develop a drop of blood. The first drop of blood was wiped off using a sterile cotton swab to avoid contaminant with interstitial fluid. When the second drop of blood had developed, the test strip for each meter was touched to the blood drop until the unit meter beeped. Different testing fingers were used for each finger stick. All samples were handled under Universal Precautions.

Participants completed 2 testing sessions. The second testing session occurred within a minimum of 48 hours and a maximum of 2 weeks after the first testing session, and the order of the sessions was randomized. Each participant completed a testing session using the pneumatic BFR Tourniquet System during the exercise protocol (EXP) and completed a second testing session using the same protocol without the pneumatic BFR Tourniquet System (CON).

The standardized blood draw protocol was used to obtain PRE blood draw samples. After resting blood samples (PRE) were obtained, proximal arm and proximal thigh circumferences were measured to determine the appropriate tourniquet band size for each participant. Participants then completed the randomly assigned EXP or CON exercise session. During EXP, tourniquets were applied bilaterally on the proximal arm and proximal thigh and inflated to pressures recommended by the pneumatic tourniquet software for a healthy individual at a hard intensity level. The software recommends occlusion pressures for the upper and lower extremities based on age, sex, activity level, and desired intensity of occlusion. Participants completed the CON session with the same exercise protocol without the tourniquets.

Exercise protocols were completed on the VersaClimber SM (VersaClimber, Santa Ana, CA). VersaClimber SM was chosen as we were occluding all 4 extremities and the machine requires the use of all 4 extremities during exercise. With the resistance provided by the machine, there is both an aerobic and anaerobic response elicited by typical use of this machine. We felt this would give an appropriate stimulus to determine the systemic cellular response after the exercise session. Participants completed 3 sets of 3 minutes of exercise on the VersaClimber separated by 1-minute rest periods. Participants were instructed to maintain a loose hand grip, avoid a static squatting position during climbing, avoid hanging on the arms, and maintain full use of the lower body throughout the climbing bout.^24^ During both rest periods of EXP, tourniquet pressures were checked and readjusted to the recommended pressure if needed. Recommended pressures varied based on the tourniquet software factoring in subject specific variables such age, sex, activity level, and desired occlusion. The Borg^25^ rating of perceived exertion (RPE) was recorded every minute of exercise. The Borg scale is quantitative measure of perceived exertion using in exercise studies. The scale ranges from 6 to 20, with 6 being no exertion and 20 being maximal exertion. The 9-minute exercise bout was terminated early if the participant reached failure (RPE = 20). Total accumulated exercise time and number of stairs climbed were recorded. Immediately after the exercise protocol, an additional 6 mL of venous blood was collected for POST. Fingersticks were performed to assess blood lactate and glucose. The remaining condition (EXP or CON) was a repeated on a second testing day with ≥48 hours of recovery between sessions. The change in cellular components was found at the PRE- to POST-interval blood draws.

Repeated-measures analysis of variance (ANOVA) was used to detect differences between EXP and CON and time points for each outcome variable. Dependent variables included WBC count (K · L^–1^), platelet count (K · L^–1^), neutrophils and lymphocytes in the WBC differential (%), CD34^+^ count (cells · L^–1^), blood lactate level (mmol · L^–1^), and blood glucose level (mg · dL^–1^). Statistical significance was set a priori at *P* < .05. Two (session) × 2 (time) repeated-measures ANOVA was used to detect differences between EXP and CON sessions of PRE and POST for all dependent variables. All analyses were performed using IBM SPSS Statistics version 24.0 software (IBM, Armonk, NY).

## Results

The mean time between testing sessions was 8.1± 4.7 days. Two participants reached maximum RPE before completion in both the control and experimental sessions. In the control session, 1 participant went 2 minutes longer than the experimental session, and the other went 3 minutes longer without the tourniquets. All other participants completed the full 9-minute protocol. The mean RPE for the experimental session was 17.6 ± 2.7 and 16.4 ± 3.2 for the control session (*P* = .01).

The mean Tegner activity level for the participants was 5.5 ± 0.9 (Table 1). A significant increase from PRE to POST exercise values was observed in both the EXP and CON groups with respect to WBC counts (*P* < .001), platelets (*P* < .001), lymphocytes (*P* < .001), CD34^+^ (*P* < .001), and blood lactate (*P* < .001) (Table 2). Conversely, a significant decrease in peripheral neutrophils (*P* < .001) from PRE to POST exercise after both the experimental and control sessions was observed. Despite the increases noted at POST in both the EXP and CON exercises, respectively, repeated-measures ANOVA revealed no significant difference between EXP and CON group values for any of the variables. There were no differences in blood glucose levels between PRE and POST for either session (Table 2).

**Table 2:** Results of the Cellular Analysis

Variable	Experimental		Control	
	Before	After	Before	After
WBC (K · L^–1^)	7.8 ± 1.4	11.8 ± 2.5∗	7.5 ± 1.8	11.3 ± 3.0∗
95% CI	7.0, 8.5	10.3, 13.2	6.5, 8.5	9.5, 13.0
Δ from before (%)		51.3		50.7
Platelets (K · L^–1^)	258.6 ± 52.5	309.9 ± 52.7∗	263.1 ± 44.7	316.1 ± 43.9∗
95% CI	228.4, 288.9	279.5, 340.4	237.3, 289.0	290.8, 341.5
Δ from before (%)		19.8		20.1
Neutrophils (%)	56.8 ± 6.6	49.0 ± 9.8†	52.1 ± 5.6	45.2 ± 6.5†
95% CI	53.0, 60.6	43.4, 54.7	48.9, 55.3	41.4, 48.9
Δ from before (%)		–13.7		–13.2
Lymphocytes (%)	32.4 ± 6.6	39.8 ± 9.8∗	36.2 ± 5.5	43.2 ± 6.7∗
95% CI	28.6, 36.3	34.1, 45.5	33.0, 39.4	39.3, 47.0
Δ from before (%)		22.8		19.3
CD34^+^ (cells · L^–1^)	3.1 ± 1.6	4.3 ± 1.8∗	3.3 ± 1.9	4.4 ± 1.4∗
95% CI	2.2, 4.0	3.3, 5.4	2.2, 4.4	3.5, 5.2
Δ from before (%)		38.7		33.3
Lactate (mmol · L^–1^)	1.8 ± 0.8	10.7 ± 3.9∗	1.7 ± 0.7	9.9 ± 3.2∗
95% CI	1.3, 2.3	8.5, 13.0	1.3, 2.1	8.0. 11.7
Glucose (mg · dL^–1^)	105.4 ± 19.8	108.4 ± 14.2	102.6 ± 18.8	96.1 ± 9.5
95% CI	93.9, 116.8	100.2, 116.5	91.8, 113.5	90.7, 101.6

CI, confidence interval; WBC, white blood cells.

∗ Significant increase from before.

† Significant decrease from before.

## Discussion

The most important finding of this study was a significant increase from baseline in CD34^+^ markers after exercise in both the EXP (38.7% increase) and CON (33.3% increase) sessions. However, despite the greater increase noted in the EXP group, there was no statistically significant difference between the overall increases in the 2 groups. A significant increase in peripheral platelets after exercise in both groups is consistent with previously published literature demonstrating a general rise in peripheral HPCs after standard non-BFR exercise.^26^, ^27^, ^28^, ^29^ The significant lactate elevation noted immediately after exercise is consistent with previously published findings and indicates that the participants were exercising at a high enough level to cause a desired systemic metabolic response.^14^, ^15^, ^16^, ^17^, ^18^

An emerging area of interest in orthopedics is to use exercise both with and without BFR to potentially optimize point-of-care blood products.^9^^,^^10^^,^^26^ BFR may be potentially leveraged as a way to non-invasively increase peripheral platelet release before blood draw to improve the platelet-rich plasma (PRP) yield. The overall higher average platelet count noted in the EXP group should be taken into consideration if one wishes to alter the components of a point of care blood product.^26^ Previous literature has demonstrated variability in platelet product yield among commercially available PRP kits.^30^ The rise in platelets in the EXP session was consistent with recent findings showing an increase in peripheral mobilization of platelets after vigorous exercise.^9^^,^^10^^,^^26^ These studies, however, focused on traditional training methods not using BFR, which could explain the similar yet significant platelet elevation (19.8% vs. 20.1%) noted in both the EXP and CON sessions.^26^, ^27^, ^28^, ^29^ Additionally, it is important to consider individual variability in blood levels as well as the variability in blood levels at different time points in the same individual.

Anz et al.^26^ found that 20 minutes of vigorous exercise increases platelet concentration by >20% in PRP products, and buffy coat–based PRP prepared after exercise had significantly higher concentrations of mobilized hematopoietic progenitor cells. Callanan et al.^10^ recently reported significant elevations of CD34^+^ cells and platelets above control values immediately after an exercise session that included 4 sets of 30-15-15-15 repetitions for the seated leg extension, prone hamstring curl, and semi-reclined leg press using the Delfi PTS Personalized Tourniquet System. Their results suggest that a statistically significant mobilization of hematopoietic progenitor cells (72% vs. 4.3%) and platelets (14% vs. 4.9%) to the peripheral circulation occurs with BFR, beyond that of the control session.^10^

Lymphocytes and neutrophils were examined in this study, as we hypothesized that these cells could potentially represent indirect markers for the peripheral release of stem cells. There was a significant increase in lymphocytes and, conversely, a significant decrease in average neutrophils immediately following exercise in both the EXP and CON sessions. We speculate that the significant rise in lymphocytes and the decrease in neutrophils may represent the release of progenitor cells that were registered as lymphocytes by the automated processing that was used for the CBC analysis. We did not, however, observe a similar mobilization of hematopoietic progenitor cells or platelets using the pneumatic tourniquet system for full-body aerobic exercise on the VersaClimber. Possible explanations for this would be that the exercise with the VersaClimber focused more on aerobic exercise versus pure resistance training. Another possible reason for the difference noted in this study would be that the tourniquet system used did not have as great or consistent of an effect on occluding blood flow compared with the Delfi system to elicit a similar systemic response. These 2 factors, both the unit specifics and the selection of BFR exercise, should be taken into consideration to manipulate point-of-care products.

The tourniquet system used in this study has a 5-cm cuff width and a detachable pressurizing system allowing for multiple cuffs to be inflated at the same time and does not restrict the participant to a certain area, but because of the use of lower pressures, this system can be more tolerable to the user during exercise than electronic systems. Furthermore, unlike electronic systems that carry a significant financial burden and are cost prohibitive, this can be an affordable alternative system. As previously mentioned, future studies should continue to investigate the influence of differing training modalities using BFR on platelet and HPC release. Ideally these results could also be compared across other commercially available BFR systems to further determine the optimal training method and system to achieve the most desirable systemic metabolic response. Further investigation should also be undertaken to identify and delineate whether there are any patient-specific factors that may correlate with a greater mobilization of platelets and HPCs after exercise with BFR over standard exercise. This would further allow for determination of who may benefit most from exercise with BFR for rehabilitation purposes, as well as if there is any potential for leveraging point-of-care blood products, something we did not show with this specific system.

### Limitations

This study is not without limitations. This exercise used focused on a systemic anaerobic/aerobic cardiovascular workout with the VersaClimber versus traditional weight-training exercises. In addition, males and females were included in the study sample, and although they were equally distributed, the role of sex on the metabolic response to exercise could also be a factor to consider. This number was secondary to the selection criteria, as well as the fairly invasive nature of the study. The use of manual differentiation of the CBC for post-training blood draws versus our automated processing may also have potentially clarified some of the significant changes noted, specifically the elevation of lymphocytes and, conversely, the significant decrease in average neutrophils. Lastly, we did not directly measure muscle oxygen tension to determine any significant differences that existed between the occluded and regular exercise sessions.

### Conclusions

There were no significant differences in systemic cellular responses when undergoing aerobic-based exercise with and without a pneumatic tourniquet system.

## Supplementary Data

ICMJE author disclosure forms
